# Responsibility driven learning in primary care: a qualitative evaluation of a medical student COVID-19 volunteering programme

**DOI:** 10.1186/s12909-022-03805-w

**Published:** 2022-10-26

**Authors:** Samantha Coster, Ravi Parekh, Zoe Moula, Sonia Kumar

**Affiliations:** Medical Education, Innovation and Research Centre, Department of Primary Care and Public Health, Imperial College London, London, UK

**Keywords:** Volunteering, Primary care, Medical students, Service-learning, Pandemic

## Abstract

**Background:**

During the first wave of the pandemic when clinical placements were suspended, a UK medical student volunteering programme was developed to support local GP practices. This study aimed to explore the impact that volunteering in primary care had on students’ learning and professional development to inform the design of future service-learning curricula innovations.

**Methods:**

Seventy medical students across all years volunteered across forty-five GP practices in north-west London. Ten volunteer students and six GPs who had hosted students volunteered to participate in remotely conducted, semi-structured interviews with a researcher. Transcriptions were independently coded by two researchers and analysed by thematic analysis using service learning and communities of practice as sensitising concepts.

**Results:**

Analysis showed a strong alignment between the views of students and GPs in terms of perceived learning. Our analysis of both sets of interviews resulted in five themes describing student outcomes from the volunteering scheme: developing as a doctor, understanding the complexity of medicine, responsibility driven learning, a meaningful role in a community of practice, and seeing behind the scenes in primary care.

**Discussion and conclusion:**

Results from this study highlighted how a meaningful service-led role and responsibility in primary care can empower and motivate students to learn beyond the traditional medical curriculum and assessments. Adopting these new ‘pro-active’ roles within general practices led volunteers, particularly those in the early years of study, to develop a better understanding of primary care and medical complexity. It also enhanced their professional skills, attitudes and behaviours, while having a beneficial impact on patient care during the pandemic.

## Background

The emergence of COVID-19 challenged health services globally. In response, medical schools across the world developed medical student volunteering programmes within secondary and primary care [1, 2]. Following the establishment of a volunteering programme by the Primary Care and Public Health department at Imperial College London during the first pandemic lockdown, this qualitative evaluation aimed to explore (i) the experiences of student volunteers in general practice (ii) whether the roles had an impact on students’ learning and professional development and (iii) the key processes behind any learning outcomes to inform service-learning opportunities.

Previous international surveys and reports suggest that medical student volunteers were primarily motivated by a desire to help their communities and support their profession [3–7] However, students recognized that volunteering provided an opportunity to gain healthcare experience when on-site clinical placements were suspended (8). Clearly, working within healthcare teams or ‘communities of practice’ with the shared and unified goal of contributing to the national pandemic effort had the potential to develop students’ expertise and confidence through a process of “legitimate peripheral participation” [8, 9]. Equally, for many students, volunteering provided them with a novel service-based role which may have afforded them a different perspective of the healthcare system, encouraging a transformative shift in their learning [10, 11]. However, these learning opportunities may have been limited by the fact that volunteering programmes were established at pace to provide critical support to stretched healthcare services, and thus they were unstructured, with no pre-defined student learning objectives and no formal process for critical reflection.

It is important to understand the experiences and outcomes for volunteers during this unprecedented time, to ensure the development of clinically appropriate, worthwhile future volunteering roles and also to inform the design of in-curricula service-based learning. However, previous studies during the COVID-19 pandemic have tended to focus on the attitudes, barriers, and facilitators to medical student volunteering [3–5, 12, 13]. The small number of studies which have examined volunteering outcomes have been largely confined to the acute sector [14–18]. Our study explores the experiences of students who volunteer within primary care and the unique learning opportunities that this setting brings. In contrast to many previous studies of volunteering during the COVID-19 pandemic, we adopted a qualitative approach, interviewing volunteers and supporting GPs to understand both the impact that volunteering had on students and the processes or factors which they perceived had led to these outcomes.

## Context

MBBS students at Imperial College London from all years were given the opportunity to sign up for GP based volunteering to assist overwhelmed practices during the first wave of the COVID-19 pandemic. Undergraduate Primary Care Education (UGPCE) matched students to general practices in north-west London who expressed an interest in hosting volunteers. The students could volunteer for up to 10 hours a week to avoid disrupting their online teaching. Appropriate duties for each student year group were drawn up based on the UK Medical School’s Council recommendations which reflected students’ experience. Support was provided by the on-site GP supervisor within the practice, and through small group tutorials run by UGPCE. Student duties were low-risk, remote patient activities such as administrative tasks, supporting vulnerable and shielding patients by telephone, and delivering medicines. In total, seventy medical students from all years volunteered across forty-five GP practices. Students volunteered for between two weeks and three months during April to September 2020.

## Methods

Semi-structured interviews were held with both student volunteers and GPs from practices who had hosted students. Interview schedules were designed to elicit students’ experiences and the impact of volunteering on their learning and development. GPs were also asked about their reflections on hosting students and on their perceptions of student learning during the volunteering process. All students and GPs were invited to participate in the interviews on completion of volunteering and all who accepted the invitation were interviewed. Seven students from years 1–3 and three from years 4–6 were interviewed between May and September 2020. Six GPs were interviewed at the end of the scheme in September 2020. After ethical permission was gained, all interviews were conducted remotely by a researcher (SC) who was not part of the volunteering programme organisation.

### Data analysis

As the study aimed to explore both the experiences and unexpected outcomes of volunteering, we adopted an open, interpretative approach, with the theories of communities of practice and service learning used as sensitising concepts at the start of the analysis [8, 9, 11]. Interviews were transcribed verbatim and imported into Dedoose (version 8.4.43). Thematic analysis was chosen as a flexible approach to analysing the data, untied to a particular theoretical stance, allowing the examination and comparison of different interviewee perspectives, and facilitating the discovery of potentially unexpected outcomes [19].

To ensure credibility, all members of the research team were involved in the six-step process of analysis (familiarisation of the data, generating initial codes, development of themes, reviewing themes, defining themes and thematic presentation for the final report.) Firstly, two researchers (SC and ZM) independently reviewed a selection of the data to develop initial codes. They discussed the initial coding to identify areas of disagreement and to reach consensus. The rest of the transcripts were then coded, and through an iterative process, the same researchers identified and discussed the emergent themes and sub-themes. For the final three steps, excerpts of the transcripts were reviewed by SK and RP to further define themes and to establish how well they reflected the narrative of the overall dataset. Themes from the data are presented in the paper with quotes to show that the content and described meanings are consistent.

The researchers ensured reflexivity throughout by being mindful of how their backgrounds and professional roles might influence data collection and subsequent analysis. The coders comprised two health/education researchers who were unconnected to the volunteering scheme with no prior educational responsibility for students (SC and ZM). The two other members of the team were academic GPs (SK, RP), one of whom had acted as supervisor to several volunteers and therefore likely to have views about the volunteering scheme.

## Results

There was significant alignment between the reflections of the students and the GPs in terms of the impact of volunteering and the factors facilitating learning. Our analysis of both sets of interviews resulted in five themes describing student outcomes from the volunteering scheme: developing as a doctor, understanding the complexity of medicine, responsibility driven learning, a meaningful role in a community of practice, and behind the scenes in primary care.

### Developing as a doctor

The task most undertaken by students in this sample was telephoning patients who were identified as shielding or vulnerable, to ensure that patients were coping and had access to the services that they needed. Although these calls were initially *“daunting”* for some students, in time they valued this unique opportunity to connect independently with such a diverse range of patients, with freedom to explore their own ways of developing communication skills and rapport:

*“Yes, you need stuff to get down to and make a diagnosis and all that, but having to just strike up a conversation is also quite an important ability. It’s just all part of building a rapport, a relationship with the doctor.”(S9, Y4)*

As students tried to employ learning from their communication skills training, they commented on the challenges of connecting with patients by telephone:

*“You always get taught a lot about body language and you smile and make them feel comfortable. But over the phone you can’t really do that.” (S2, Y3)*

Particularly for early years students, contacting vulnerable patients also required a degree of emotional regulation, to enable them to find a way of working with their own discomfort in the face of patient suffering:

*“To have a 45-minute call with someone who’s really upset, bordering on tears and then ending that call, and then having your next one on the list, I had to take a break. I found that hard. In the end, I just had to write up the notes, file it and try and get on with the next one.” (S5, Y1)*

Building their skillset by careful observation of other practice staff and reflection on their own successful and less successful patient contacts, they began to feel a sense of agency in the way that they managed their calls. GP supervisors also believed having independent conversations with patients had developed their ability to build rapport remotely and had helped them to start to find their professional voice:

*“I think he [the student] learnt to listen. And I think he also learnt to find his voice that he would develop as a future consultor — as a doctor. And also had the freedom to experiment a bit with how he asked questions with no one judging him. Like a little private apprenticeship.” (GP6)*

### Understanding the complexity of medicine

The nature of their conversations with patients focused not just on their medical care, but also on their social support, emotional needs and their environment. Most GP interviewees agreed that hearing patients’ personal narratives of coping in the pandemic had provided students with a better understanding of health within a social context. GPs felt that volunteers had been exposed to the complexity of managing patients in general practice, including not *“just the clinical side of looking after patients, but the social side of looking after them” (GP3*) and that the pandemic had brought this into focus:

*“I suppose that the whole COVID shielding is a completely new bit of medicine that none of us have learnt about. Sure, they were shielding because of their asthma, but then you need to think who’s going to get their shopping and all that kind of stuff which we have to think about as a GP and it is stuff that doesn’t get taught in medical school really.” (GP1).*

Such conversations prompted students to reflect on the challenges of looking after vulnerable and chronically unwell patients, but also on the role of the doctor. Students could see both the limitations but also the potential of the doctor’s role:

*“You just see a person — you don’t really see their life outside of hospital. Whereas when you phone them you really understand how differently they all live and the things that they all have going on in their lives. And some patients might have more debilitating disease than others, but they might have very vast support systems.” (S2, Yr3)*

*“I was thinking about how holistic care needs to genuinely provide some sort of substantial change in a patient’s life. There are so many other things going on in their lives that mean they can’t put in that time or can’t truly recover properly …. so, yes, the importance of a support system and how you as a doctor can be that best point of support.” (S8, Yr3)*

## Responsibility driven learning

Students were aware of the responsibility attached to their volunteering role.

*“As far as the rest of my experience goes, [it’s] probably the most responsibility I’ve had in a healthcare environment.” (S3, Y2)*

They described using their initiative to make independent decisions about how to support patients, and how over time this fostered their self-efficacy. For early years students in particular, the responsibility of volunteering felt challenging, but also helped them to develop a degree of resilience:

*“I think that entire experience, of being pushed out of my comfort zone, was very uncomfortable at the time but definitely rewarding — realising that there were some mistakes that I made, and that some of the mistakes weren’t mistakes, in the sense that nothing bad happened as a result of what I’d done. It really helped me feel a little bit more comfortable going into the next year of medical school.” (S6, Y1)*

Conversely, some later year students who were more clinically confident felt that they could have taken on more responsibility and were frustrated with the safety limitations of their roles. Those students who had less responsibility than they had hoped for, fewer patient-facing tasks, or who volunteered towards the end of the first pandemic wave when the sense of urgency had decreased, tended to be less engaged and stopped volunteering sooner.

*“Especially when they said it was a three-year backlog of notes and I thought that that really wasn’t related to COVID. I was actually in an office of five people all doing admin stuff. That’s why in the end I felt a bit — I don’t think I’m really needed here.” (S10, Yr 4)*

GPs explained how the volunteers had relieved the pressure on their time and on the time of other members of staff which allowed them to better utilise their practice resources. They described how students had provided a *‘clear-headed advisory service,’* having responsibility for registering patient concerns, advising, reassuring, and importantly signposting patients to relevant services. They also believed that while taking responsibility had clear benefits for students, it had also been a vital resource for patients, with volunteers being an important bridge between the patient and GP during the pandemic:

*“In terms of patients, they certainly appreciated the call. I think they were all feeling that hospitals were busy; GPs were busy and they didn’t really want to be calling us about little things that might have been on their minds. It turned out some of those little things were actually quite big things.” (GP3).*

### A meaningful role in a community of practice

Whilst some students had minimal contact with other practice staff and felt limited attachment to their place of work, other students were placed at the centre of busy teams and invited to attend team meetings and social events. These students experienced a strong feeling of community within the GP practice:

*“We were encouraged to join in on the morning meetings, the top of the day, group meetings with the entire GP staff — it was also part of what made it feel like I was part of the team, like I was part of the rest of the staff.” (S6,Y1)*

Students reported working alongside reception and administrative staff, nurses, pharmacists and social prescribers. Although students were keen to learn more from other staff, they were also concerned about being a burden rather than a help during pressurised times. Despite this, many reported interprofessional learning through actively supporting staff, and by listening to the communication occurring between different parts of the practice:

*“So, I was working with physician associates, so I’d work quite closely with them. I would work with the GPs, with pharmacists, with reception staff, practice managers. I probably never would have done that before.” (S4, Y4)*

Some GP supervisors maximised students’ learning by discussing the clinical management of patients that they were phoning, encouraging students to sit in on remote clinical consultations, and involving students in audit and research projects. These GPs explained how they had tried to increase the value of the role to the student and saw volunteering as a *‘two-way street’*:

*“We made sure they sat in clinic consults so they can get clinical exposure just so that they could learn as well. Because there was so much learning. There’s no point in being in a GP centre and not learning all the medicine as well.” (GP6).*

### “Behind the scenes” in primary care

Accepted as part of the practice, students were privy to ‘behind the scenes’ conversations that they would not have witnessed on placement. Working alongside non-medical staff led to a greater appreciation of the skill required for these essential roles. Students reflected on how volunteering had changed their perceptions of general practice. For some, GP work was not as solitary as they had imagined, and they became more aware of the importance of teamwork to ensure everything ran smoothly. Students also became aware of the wide scope of consultations in primary care:

*“I mean, reading through the patient’s notes as well gave me a little bit of insight on what the GP consultations actually consist of. It’s not necessarily just, ‘I have an illness can you help it?’ There’s also a lot of counselling involved as well and that really showed through the patients’ notes.” (S3, YR2)*

Some GPs also made efforts expose their volunteers to some of the business and managerial aspects of running a practice, with which most were unfamiliar.

*“Because they were with me all the time and it was what was happening, they got to see what we have to do. Our actual job. Not just the clinical side, but the rest of the job, which is all encompassing. I think that was good exposure for them to see the inside workings of a primary care team.” (GP6)*

GPs hoped that volunteers left with a better idea of how general practices really worked, and that this might help them as doctors to work in or with primary care in the future:

*“Now understanding the level of detail, you have to go into. The way we have to work, so that when they are in secondary care, they make safe discharges. They can now have good conversations with their primary care colleagues …. and they wouldn’t get that from a standard, two-hour tutorial.” (GP5)*

### Implications for future practice

Interviewees were asked directly whether there were aspects of the volunteering role which might be useful to consider when developing service-led medical curricula. Both GPs and volunteers argued for introducing similar clinical responsibilities earlier in their studies. Students believed that talking to patients independently but remotely would provide an opportunity for them to develop communication and rapport. GPs also mentioned fostering interprofessional student learning by forging stronger links between medical students and social prescribers, link workers and reception staff. Finally, GPs praised the volunteers’ “can do” approach having encountered students with less flexible attitudes on placement who were solely focused on observing the GP in clinic. Both students and GPs felt that this had broadened their experience of general practice and widened their understanding of healthcare.

## Discussion

The majority of interviewees reported that their primary motivation was to assist their profession and the community, but some students also wanted to utilise their time and skills constructively and gain more experience within healthcare settings. Most of those interviewed did report learning beyond their *“curriculum and exam material”* with wider professional skills development. There has been voiced concern that unstructured volunteering may be detrimental to student learning [20]. However, our research supports the small number of studies from COVID-19 volunteer schemes in acute settings where medical students reported the acquisition of valuable skills [14–18]. Importantly, GPs also reported that students had a significant impact on clinical care by acting as a vital bridge between the GP and their patients, as they took up these newly adopted ‘para-professional roles’ [21].

Previous studies of service-learning suggest that all students learn most effectively when they perform real-work tasks, are in a position of responsibility and ownership, and are supported by appropriate supervision and mentorship [11]. Our data suggested that volunteers thrived in practices where they were not only given responsibility, but where they were also valued and embraced as part of a ‘community of practice.’ Some staff invested their own time to support students, to mentor them and to include them in the everyday business of the practice, even though this was not a requirement of hosting. This mirrors the significant influence of staff engagement on the quality of service-learning opportunities offered to students within the standard curriculum [22, 23].

Students valued the opportunity to have independent “conversations” with patients which were different to placement consultations. Unique perhaps to primary care volunteering, students were not always caring for patients who were acutely unwell. Instead, they were learning how to connect with people from different cultures to address potentially sensitive issues, and how to build this rapport remotely. Such challenges are useful for developing a skillset for telephone consulting which is becoming more commonplace in general practice and other sectors [24]. Without having to address immediate health issues, students instead spoke with patients about their mental wellbeing, financial situation, bereavement and social isolation. Hearing patient tell their stories fostered reflections in early years students which transformed their appreciation of both the role of the doctor and of social medicine. Our study supports research which shows that early patient contact increases students’ awareness of professionalism [25, 26] and that discovering more about patients as individuals can encourage patient-centred attitudes [27]. Volunteering provided students with a greater opportunity to see the “person behind the patient” which gave further insight into the huge impact of the pandemic on vulnerable individuals.

Previous studies of medical student volunteering in acute care during the pandemic suggested that having responsibility strengthened students’ emotional development, challenged their resilience, and developed their stress management skills [16, 17]. In this study, being entrusted with decisions about how best to manage patients’ needs felt significant to early years students and did appear to both drive their learning and build their resilience. Later years students and those with no patient-facing tasks tended to be less engaged and reported less personal benefit from volunteering. Some later years students reported feeling restricted by safety guidelines and had hoped for more responsibility considering their prior clinical experience. By contrast, a study in acute care found that senior student volunteers were more anxious about “working outside their competencies” and of the extra burden that poor performance might place on their clinical team. Whilst early years students with lower expectations were surprised and positive about the impact that they were able to have in their volunteering role [14].

This remote-consulting model based in primary care, eliminated most of the worry of a physical risk from COVID-19 and limited psychological distress. Although listening to patients’ struggles was sometimes upsetting, our interviewees felt able to find support from either practice members or faculty when needed. Equally, the exposure to the affective components of medicine within general practice clearly offered the volunteers huge learning opportunities, revealing first-hand the challenges of showing compassion while protecting oneself, a skill not often covered or experienced as a medical student.

The volunteering role also provided students with a different perspective on the workings of primary care, and its complex relationship with secondary and social care. Improving students’ understanding of the breadth of general practice, the intellectual challenges it affords, and importance of the various business elements, were flagged by the Wass report [28] as key to raising the profile and status of general practice among medical students. This broader learning occurred largely because student roles were not assessed or formally aligned with the curriculum, freeing them to *“get stuck in”* all aspects of practice life. Hosting GPs contrasted this with their observations of clinical placements where students can feel forced to limit themselves to just shadowing clinic appointments. Thus, it may be worthwhile to find ways of encouraging students to become more flexible and pro-active on placement to maximise their time in general practice.

Previous studies in acute care report that volunteering provides students with a better understanding of the roles of non-medical professionals [17, 18]. Our volunteers were also struck by the importance of interprofessional team working in primary care and were often surprised to find that GPs do not work in isolation. Relatedly, our study demonstrates how students can be supported not only by GPs, but also by reception staff, nurses, social prescribers and other staff members. This also brings into focus the importance and value of involving the full practice team in developing curricula-based service-learning. Given that they will have potentially less experience of supervising medical students or assessing their capabilities, it is also necessary to ensuring adequate support for these staff, such as those in reception, who will pass on key ‘on the ground’ skills in communication and negotiation with patients.

## Strengths and limitations

This is one of the first papers to use an exploratory approach to examine the impact on students who volunteered in primary care during the COVID-19 pandemic and to have invited both GP supervisors and students to provide reflections on their experiences. However, interviewees were from a self-selected pool of volunteers and supervisors and thus their views and experiences may have been different to those who did not agree to be interviewed. In addition, given the important role that non-medical members of the primary care teams played in the volunteering process, the research would have been strengthened by the inclusion of these members of staff in the interview sample.

## Implications and conclusions

Medical students who participated in this study had largely positive experiences of volunteering in primary care, and early years students in particular reported the development of a range of skills which they perceived as valuable to them both as medical students and future doctors. This study demonstrates that engaged practice staff, who are genuinely interested in developing their students, are key to successful learning experiences. The experience of participants also highlights the value of forging stronger links between medical students and non-medical members of the interprofessional primary care team, while ensuring that such staff are adequately supported to teach or supervise medical students. Indeed, future research should examine the role of the interprofessional team during undergraduate placements in primary care in order to help maximise their potential contributions to student learning.

It is also worthwhile considering ways to entrust early years students with appropriate patient responsibility, such as supporting those who are housebound to perhaps review their medication or to check on their wellbeing. Such tasks can be safely facilitated by using workplace-based assessment tools, such as entrustable professional activities [29]. Not only could such activities be of benefit to under-resourced practices, but it may aid the development of students’ communication skills and their understanding of medical complexity. More broadly, the study also suggests that students should be actively encouraged to embrace the range of opportunities presented during their general practice placement. Such an approach will undoubtedly help students embed more authentically into their host practices, promoting greater informal learning from staff whilst also broadening their knowledge of primary care.

Our study showed that students became active participants of the GP ‘community of practice’ contributing valued and much needed service at a time of national need. When medical students in the earlier years were actively engaging in service-led tasks in general practice, responsibility, not assessments, was the primary driver to learning. Such approaches in formal medical curricula, have the potential to benefit patient care, and impact on students’ acquisition of knowledge, skills, and professional values and attitudes.

## Data Availability

The datasets generated and analysed during the current study are not publicly available due to confidentiality considerations as stated by the ethical approval process, but may be available from the corresponding author on reasonable request.
